# Insight into the Mode of Action of Haedoxan A from *Phryma leptostachya*

**DOI:** 10.3390/toxins8020053

**Published:** 2016-02-22

**Authors:** Zhaonong Hu, Yuzhe Du, Xinmin Xiao, Ke Dong, Wenjun Wu

**Affiliations:** 1 Institute of Pesticide Science, College of Plant Protection, Northwest A & F University, Yangling 712100, Shaanxi, China; huzhaonong@nwsuaf.edu.cn (Z.H.); xiaoxinmin@126.com (X.X.); 2 Key Laboratory of Botanical Pesticide R & D in Shaanxi Province, Yangling 712100, Shaanxi, China; 3 Department of Entomology, Michigan State University, East Lansing, MI 48824, USA; duy@msu.edu (Y.D.); dongk@msu.edu (K.D.)

**Keywords:** botanical insecticides, haedoxan A (HA), synaptic transmission, voltage-gated sodium channel

## Abstract

Haedoxan A (HA) is a major active ingredient in the herbaceous perennial plant lopseed (*Phryma leptostachya* L.), which is used as a natural insecticide against insect pests in East Asia. Here, we report that HA delayed the decay rate of evoked excitatory junctional potentials (EJPs) and increased the frequency of miniature EJPs (mEJPs) on the *Drosophila neuromuscular* junction. HA also caused a significant hyperpolarizing shift of the voltage dependence of fast inactivation of insect sodium channels expressed in *Xenopus oocytes*. Our results suggest that HA acts on both axonal conduction and synaptic transmission, which can serve as a basis for elucidating the mode of action of HA for further designing and developing new effective insecticides.

## 1. Introduction

The herbaceous perennial plant lopseed *Phryma leptostachya* L., the sole species of the family Phrymaceae, is widely distributed in the Himalayas, temperate Asia, and northern East America and is traditionally used as a natural insecticide in East Asia [1,2]. Since the 1960s, Taniguchi *et al.* [3,4,5] have isolated a series of Phryma lignans with 3,7-dioxabicyclo [3.3.0] octane skeleton, such as phrymarolin I, phrymarolin II, and haedoxan A (HA). Interestingly, HA is the sole ingredient with higher insecticidal activity against the housefly compared with commercial synthetic pyrethroids [5]. Subsequently, the synthesis and structure-activity relationships of HA were examined [6,7]. Phrymarolin I and II belong to a class of dilignans deprived of the 3-phenyl-2-hydroxymethyl-1, 4-benzodioxane framework of haedoxans and are totally inactive [8,9,10,11,12]. However, leptostachyol acetate, whose chemical structure is very similar to those of phrymarolin-I and II, is active against larvae of three different mosquito species [13] and housefly [14].

Our laboratory has systemically isolated the insecticidal ingredients of *P. leptostachya* and investigated its insecticidal activities in recent years [15,16,17]. Encouraged by the larvicidal activities against mosquitoes, four high mosquiticidal compounds were isolated, including phrymarolin I, HA, haedoxan E, and a new compound named phrymarolin B, the structure of which was elucidated as 1-hydroxy-2-(3′,4′-methylenedioxy) phenoxy-6-(2′′-hydroxy-3′′,4′′-methylenedioxy) phenyl-3,7-dioxabicyclo[3.3.0]octane [17]. The LC_50_ values of four insecticidal compounds against the fourth-instar larvae of *Culex pipiens pallen* were 1.21, 0.025, 0.15, and 0.69 mg/L, respectively. HA, haedoxan E, and phrymarolin B also have significant contact and stomach toxicities against the third-instar larvae of the oriental armyworm *Mythimna separata*. These results further illustrate that HA is the most potential insecticidal ingredient of *P. leptostachya*. Hence, developing a novel natural botanical pesticide based on HA as a major insecticidal active ingredient is promising.

Regarding the mode of action of HA, early studies have shown that when HA is ingested by larvae of some species of lepidopterous insects, HA causes early cessation of feeding, muscle relaxation and, ultimately, death, of tested insects [5]. According to the poisoning symptoms of treated insects and high stereoselectivity required for their insecticidal activities, HA may act on receptors in neurons or muscle [5,18]. Insecticidal (+)-HA and three other noninsecticidal isomers can inhibit [^35^S]*t*-butylbicyclophosphorothionate(TBPS) binding on the GABA receptor allosterically in a concentration-dependent manner by coupled interaction with the TBPS-binding site in rat brain, which is distinct from the GABA-binding site. Apparently, the lethal activity of (+)-HA against insects is not due to the action at the GABA_A_ receptor, although it may contribute to the initial excitation, as observed in the injection of haedoxan isomers into German cockroaches [18]. Therefore, the mode of action of HA remains unknown.

In the present study, we investigated the effects of HA on excitatory transmission at the *Drosophila* neuromuscular junction (NMJ) and on voltage-gated insect sodium channels expressed in *Xenopus* *oocytes*. Results showed that HA delayed the decay rate of excitatory junctional potentials (EJPs) and, increased the frequency of miniature EJPs (mEJPs) on *Drosophila* NMJ, and altered the voltage dependence of sodium channel inactivation. These findings revealed potential targets of action of HA and laid a foundation for further elucidation of the molecular mechanisms of action of this botanical insecticide.

## 2. Results

### 2.1. HA Delayed the Decay Rate of Evoked EJPs at the Drosophila NMJ

**Figure 1:**
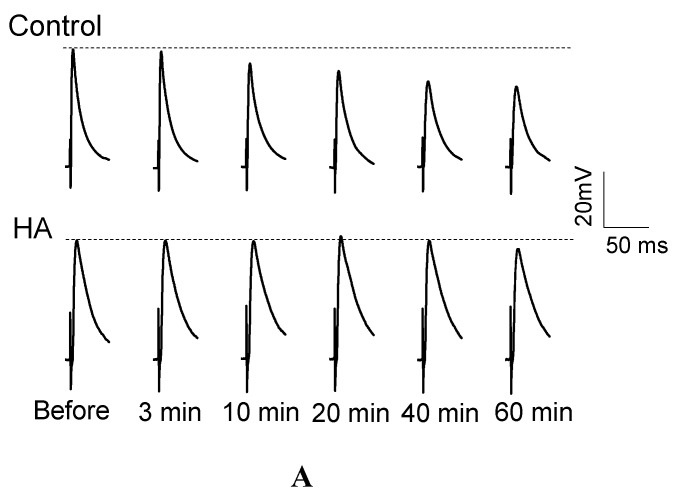

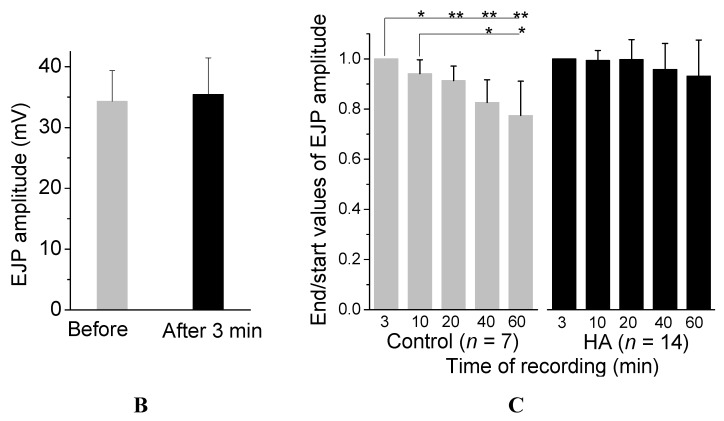
HA delayed the decay rate of the evoked EJPs at the *Drosophila* neuromuscular junction. (**A**) representative EJPs traces in control and in the presence of HA (2.5 × 10^−6^ M). (**B**) mean amplitude of evoked EJPs before and after 3 min of HA application. Evoked EJPs were elicited by stimulating the segmental nerve at 0.1 Hz with the amplitude of the pulse of 5V. The values reported are the mean ± SD. (**C**) mean amplitude of evoked EJPs at various time points (3, 10, 20, 40, and 60 min) in control and in the presence of HA were normalized to the initial amplitude of evoked EJPs, *i.e.*, *n* = 7 for the control and *n* = 14 for HA treated. Statistical significance was determined by Student’s *t*-test, and significant values were set at * *p* < 0.05 and ** *p* < 0.01.

HA causes muscle relaxation in houseflies [5,16], so we chose the *Drosophila* NMJ [19,20] to examine the effect of HA on synaptic transmission using intracellular recording. Before HA treatment in HL3 solution (containing 1.5 mM Ca^2+^), the peak amplitude of evoked EJPs was 34.32 ± 5.03 mV. In the absence of HA, the amplitude of evoked EJPs was gradually reduced in response to 0.1 Hz stimulation for 60 min (Figure 1A,C) from 94% of the initial amplitude at 10 min to 91%, 83% and 77% of the initial amplitude at 20, 40, and 60 min, respectively. Three minutes after HA application (final concentration = 2.5 × 10^−6^ M), the amplitude of evoked EJPs remained similar to that before HA treatment (Figure 1A,B). However, application of HA at 2.5 × 10^−6^ M did not cause significant decreases in the evoked EJPs amplitude from 10 min to 60 min of 0.1 Hz stimulation (Figure 1C). These results indicated that HA was able to maintain the evoked EJPs in response to sustained low-frequency stimulations.

### 2.2. HA Increased the Frequency of mEJPs at the *Drosophila* NMJ

In our recording from the NMJ of *w^1118^* flies, the average values of the resting membrane potential was 70.9 ± 1.7 mV (*n* = 50, mean ± SE). In this resting condition, mEJPs can be recorded. Each mEJP represented postsynaptic depolarization in response to the spontaneous fusion of a single synaptic vesicle [21,22].

**Figure 2:**
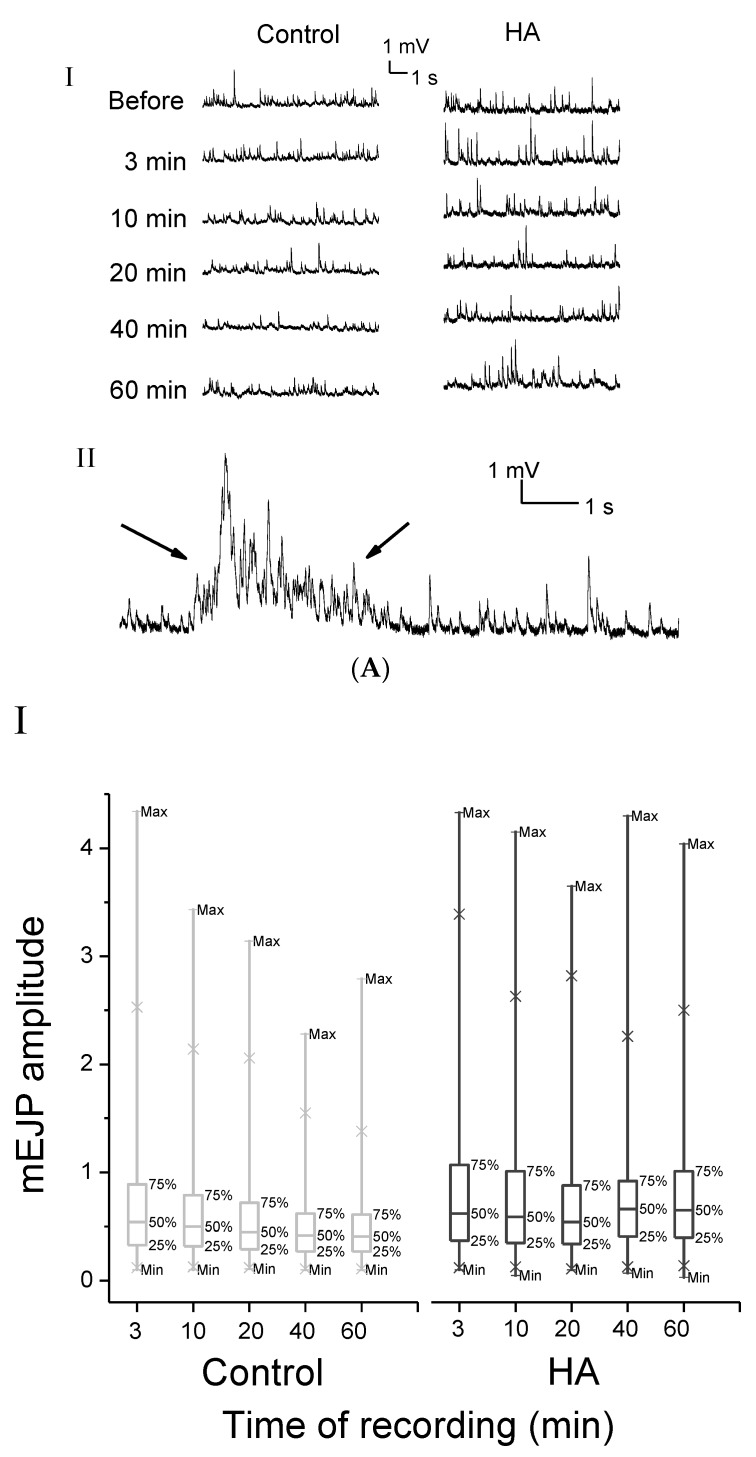

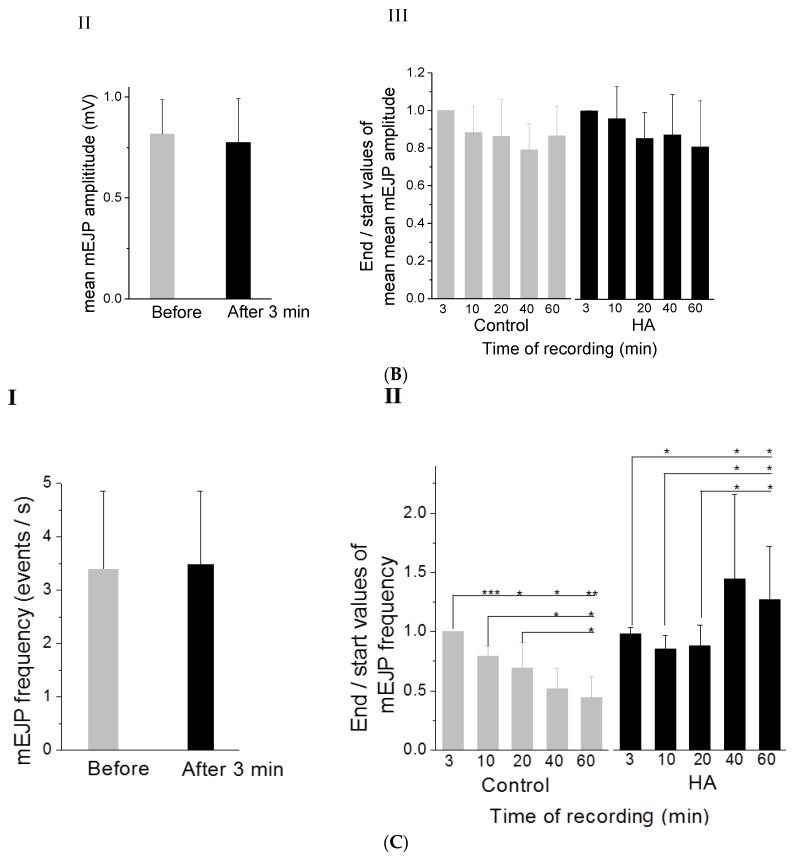
HA increased the frequency of mEJPs at the *Drosophila* neuromuscular junction. (**A**) **I**, representative of mEJP traces in the control and in the presence of HA (2.5 × 10^−6^ M). **II**, overlap and summation of mEJPs recorded 20 min after HA application, which occurred in about 33% of total recordings. Arrows indicate the irregular mEJPs. (**B**) analysis of mEJP amplitudes; HA did not affect the average amplitude of mEJPs. **I**, box plots of mEJPs amplitudes. **II**, mEJPs amplitude before and at 3 min after HA application showing no significant difference; **III**, mean mEJPs amplitude at different time points (3, 10, 20, 40, and 60 min) in the control and HA application were normalized to the initial mEJPs amplitude in the control and HA application, separately. No significant difference was observed with time on mEJPs amplitude in both control and HA application (*p* > 0.05). (**C**) analysis of mJPs frequencies, HA induced a significant enhancement in the frequency of mEJPs. **I**, mEJP frequency before and after 3 min of HA application; there is no significant difference (*p* > 0.05); **II**, mEJP frequency at different time points (3, 10, 20, 40, and 60 min) in the control and HA application were normalized to the initial mEJP frequency in the control and HA application, separately. mEJP frequency in the control showing significant decrease with time, whereas HA induced a significant increase at 40 and 60 min. Notably, the start and end values of mEJPs amplitude/or frequency in (**C**) are the EJP amplitude/or frequency at 3 min after application and other different recording times, respectively. The values reported in (**C**) are the mean ± SD. HA, *n* = 7; control *n* = 6. Statistical significance was determined by Student’s *t*-test, and significant values were set at * *p* < 0.05 and ** *p* < 0.01, *** *p* < 0.001.

At 0.1 Hz nerve stimulation, the representative of mEJP traces in control and in the presence of HA are shown in Figure 2A. The release rate of mEJPs finally increased after HA application, but that of the control decayed with time (Figure 2A-I). We also recorded the overlap and summation of mEJPs recorded after 20 min by HA application, which occurred in about 33% of the total recordings (Figure 2A-I). By box plots of mEJP amplitudes (Figure 2B-I), we noted that the median mEJP amplitude dropped slowly with time in the control, whereas the decrease in HA amplitude was similar to that of the control over 10 and 20 min. Subsequently, the median mEJPs amplitude increased at 40 and 60 min, which were higher than the initial baseline amplitude 3 min after HA application. In the absence of HA, the average amplitude of mEJPs recorded slightly decreased over the first 3 min (Figure 2B-III). Similar to that of the control, the average amplitude of mEJPs in the presence of HA also decreased over 10 and 20 min. Overall, no significant difference was observed with time for mean mEJP amplitudes on both with and without HA application (Figure 2B-II and 2B-III; *p* > 0.05). By contrast, a significant enhancement in the frequency of spontaneous miniature events at mEJPs was induced by HA (final concentration = 2.5 × 10^−6^ M). In the absence of HA, the release rate of mEJPs decreased with time. mEJP frequency dropped from 80% of the initial frequency at 10 min to 44% at 60 min (Figure 2C-I and 2C-II). Compared with the time-dependent drop of mEJP frequency in the control, the mEJP frequency in HA dropped to 0.852 ± 0.117 of initial frequency at 10 min (*p* < 0.05, *n* = 9) as same as that of the control, and then increased relatively to 0.878 ± 0.179 of initial frequency at 20 min (*p* > 0.05). Subsequently, the mEJPs frequency increased significantly to 1.444 ± 0.713 and 1.271 ± 0.451 of the initial frequency at 40 and 60 min (*p* < 0.01), respectively. Furthermore, HA can also produce some irregular mEJPs increments over 20 min, in which the mEJP rate could not be counted in most cases due to the overlap and summation of potentials (Figure 2A-II). However, the increase was usually sudden and obvious, making quantitation impossible and unnecessary. The significant enhancement in the frequency of mEJPs induced by HA indicated that HA may produce a relatively high rate of spontaneous synaptic vesicle fusion events at motor terminals.

### 2.3. HA Altered the Voltage Dependence of Inactivation of Insect Sodium Channels Expressed in Xenopus oocytes

A motoneuron carrying the impulse creates synapses with the muscle fiber, which is known as NMJs [23]. The muscle infolds participating in the synapse contains a large number of sodium channels [23]. Voltage-gated sodium channels (VGSCs) are known to play a central role in action potential generation and propagation in nerve and muscle [24]. In skeletal muscles, VGSCs are concentrated at NMJs in the depths of the postsynaptic folds [25,26,27]. The sodium current that passes through the narrow synaptic cleft affects the adjacent membranes electrical properties and in turn modifies synaptic transmission process. Hence, determining whether HA affected VGSCs was very interesting. In a previous experiment, we investigated the effects of HA on excitatory transmission at the *Drosophila* NMJ. German cockroach have been injected with haedoxan isomers to induce muscle relaxation [18], and mosquito *Culex pipiens pallen* have been chosen for bioassay. In the present work, three insect sodium channels, namely, cockroach BgNa_v_1-1a, fruit fly DmNa_v_22, and mosquito AaNa_v_1-1a sodium channels, were examined for the effects of HA on VGSCs.

**Figure 3:**
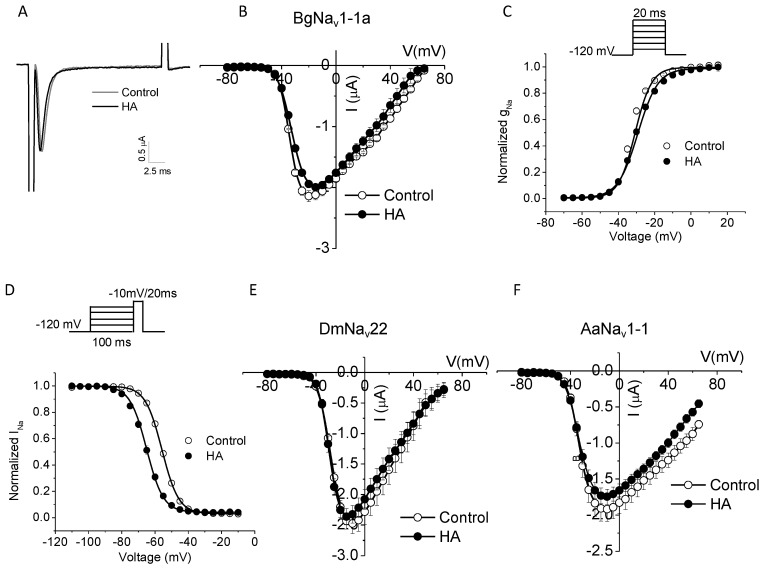
HA induced a significant hyperpolarization direction shift in inactivation-gating properties in cockroach sodium channels BgNav1-1a. (**A**) recording traces before and after the application of 10^−^^5^ M of HA; (**B**) I-V curve of cockroach sodium channels BgNav1-1a before and after the application of 10^−^^5^ M of HA; (**C**) and (**D**) the voltage dependence of activation (**C**) and inactivation (**D**) before and after the application of 10^−^^5^ M of HA. The activation and inactivation curves were fitted with two-state Boltzmann equations. The number of oocytes was 5–8; (**E**) and (**F**) I-V curve of *Drosophila* sodium channels DmNa_v_22 (**E**) and mosquito sodium channels AaNa_v_1-1 (**F**) before and after the application of 10^−^^5^ M of HA.

Cockroach BgNa_v_1-1a, *Drosophila* DmNa_v_22, and mosquito AaNa_v_1-1a sodium channels were expressed in *Xenopus* *oocytes*, and the gating properties of these channels were examined with two electrode-voltage clamp. HA did not alter the amplitude of sodium currents or the voltage dependence of activation or activation kinetics of sodium channels (Table 1 and Figure 3). However, HA (10^−^^5^M) shifted the voltage dependence of inactivation of all three insect sodium channels by 8–11 mV in the hyperpolarizing direction (Table 1 and Figure 3).

## 3. Discussion

In this study, we investigated the effects of HA on excitatory transmission at the *Drosophila* NMJ and voltage-gated insect sodium channels expressed in *Xenopus* oocytes. The mode of action of HA is studied at the molecular level for the first time. We observed that HA delayed the decay rate of evoked EJPs and increased the frequency of mEJPs on the *Drosophila* NMJ at least to 1.27- to 1.44-fold of initial frequency. We also found that HA caused a significant hyperpolarizing shift of the voltage dependence of fast inactivation of insect sodium channels by 8–10 mV. These findings meant that HA may acts on both axonal conduction and synaptic transmission. Interestingly, linking the physiological changes induced by HA in the nerve endings with its ability to modify the Na^+^ channels was essential.

The motoneuron connects to the muscle fiber at NMJs to ensure a rapid and efficient transmission of the impulse. At the presynaptic nerve terminal, the voltage-dependent calcium channels opens up increasing the Ca^2+^ ions permeability which allows Ca^2+^ to flow in. This causes the vesicles to be fused with the presynaptic membrane and release neurotransmitter at the synaptic cleft. The neurotransmitter diffuses at the cleft and binds with the relative neurotransmitter receptors resulting in opening of voltage-dependent sodium channels located at the postsynaptic muscle membrane [23]. The inflow of sodium ions affects the local extracellular potential at the junction. It is difficult to test the sodium current along the synaptic cleft. Therefore, we investigated voltage-gated insect sodium channels expressed in *Xenopus* oocytes*.* However, more studies need to be done to examine whether HA induced hyperpolarizing shift of the voltage dependence of fast inactivation of sodium channels were correlated with the decay rate delayed EJPs and the increased mEJP frequency.

Electrophysiological measurements showed that HA delayed the decay rate of EJPs compared with the control. In terms of normal evoked EJPs recording, Peled *et al.* [28] reported that the amplitude of evoked EJP dropped to 0.93 ± 0.07 of the initial amplitude during low-frequency nerve stimulation over 20 min. This finding was consistent with our result, at the same time with a corresponding drop to 0.914 ± 0.057 of the initial amplitude. Based on our recording in the control, the tendency of evoked EJP drop was time dependent. By contrast, HA produced only a small, insignificant decrease in evoked EJPs amplitude, and maintained the evoked EJPs in response to sustained low-frequency stimulation. This influence of post-synaptic responses evoked by motor-nerve stimulation produced by HA could promote the stability of pre- to post-synaptic transmission.

In our recordings, mEJPs showed a shift in distribution toward lower values and a decrease in frequency with time in the control. Similar results have been obtained by Peled *et al.* [28]. In the presence of HA, although the average of mEJP amplitude was insignificantly changed, the distribution of mEJP amplitude showed a shift toward lower values and then toward relatively higher values. Compared with the control, HA interestingly produced a significant increase in frequency with time. Thus, HA may increase spontaneous miniature release or alter pre-synaptic transmission. The phenomena could result from weakened regulation of synaptic vesicle exocytosis. Rees [29] observed increased mEPSP rate and multiquantal mEPSPs after treatment of cockroach nerves with metabolic inhibitors, and these effects were correlated with the clumping of synaptic vesicles and changes in the appearance of mitochondria in the pre-synaptic terminals. Increased mEPSP rate, multiquanta1 mEPSPs, and clumping of synaptic vesicles are all believed to be due to increased levels of intraterminal Ca^2+^. When Ca^2+^ ions bound to vesicle membranes, they decreased the negative surface charge and allowed the vesicles to adhere onto one another and onto the pre-synaptic membrane, thereby, facilitating release from the cell [29]. Salgado *et al.* [30] also found that deltamethrin-induced depolarization of nerve terminals increase the rate of mEJPs in housefly, and [Ca^2+^]*_i_* changes may be the basis for the large mEJPs produced and the rate increased by the pyrethroid. In agreement with Rees and Salgado *et al.*, we proposed that HA may increase the levels of intraterminal Ca^2+^, contributing to increased mEJP release rate and multiquantal mEJPs. Therefore, further investigation of [Ca^2+^]*_i_* changes of in neuron cells is necessary to validate our prediction.

Our results also showed that HA altered the voltage dependence of inactivation of insect sodium channels expressed in *Xenopus* oocytes. HA produced a significant hyperpolarizing shift in the voltage dependence of inactivation of insect sodium channels. This finding suggested that fast inactivation developed at a more hyperpolarizing potential by HA promoting fast inactivation. Fast inactivation is known to be a highly important feature of sodium channel kinetics as it helped to repolarize the excitable membrane during an action potential “process being the repolarizing force”. In terms of modulation of sodium channel inactivation by well-known toxins, various toxins, and other chemical agents slow or even abolish inactivation [31]. Pyrethroids shift the relation between activation and membrane potential in the direction of hyperpolarization and cause a similar shift of inactivation [32]. Many drugs including local anesthetics (LA) shift the voltage dependence of fast inactivation in the hyperpolarizing direction. Spiradoline, an arylbenzacetamide antiarrhythmic drug, which also produced a hyperpolarizing shift in the voltage-dependence of sodium channel inactivation. According to the Modulated Receptor Hypothesis (MRH), such a shift can be attributed to a higher affinity of the drug for the inactive state of the channel. It suggests that most clinically useful LA and antiarrhythmic drugs have a low affinity for the resting state of the sodium channel. Selective, high-affinity binding of the drug for the inactive state of the channel imparts specificity of blockade to abnormally-firing sodium channels, without inhibiting normal neuronal and cardiac sodium channel function [33]. Therefore, like LA, HA may have high-affinity binding for the inactive state of the sodium channel. In addition, dietary polyunsaturated fatty acids (PUFAs), particularly, *n*-3 class as contained in fish oil, are antiarrhythmic agents. They exerted their effect on cardiac (Na_v_1.5) Na^+^ channels by reducing I_Na_ and shifting the steady-state inactivation curve in the hyperpolarizing direction [34]. Pyrethroids and PUFAs significantly shifting the steady-state inactivation to more hyperpolarized direction may be similar to HA, but HA did not alter sodium current amplitudes and the activation kinetics of sodium channels. Further investigation on sodium channel inactivation will be conducted in the future.

## 4. Materials and Methods

### 4.1. Compounds

**Figure 4:**
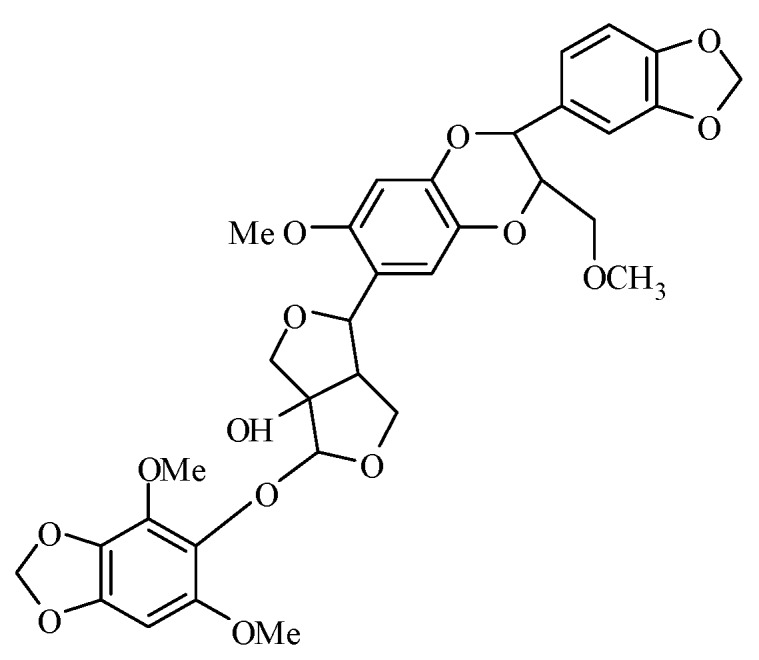
Structure of haedoxan A.

Haedoxan A (HA; technical grade, ≥98%) was provided by the Institute of Pesticide Science, Northwest A & F University (NWAFU, Yangling, Shaanxi, China). The structure of haedoxan A was elucidated as l-hydroxy-2-[(2,6-dimethoxy-3,4-methylenedioxyphenyl)oxy]-6-[6′-methoxy-2′-methoxymethyl-3′-(3,4-methylenedioxy)phenyl]-2′,3′-dihydro-l′,4′-benzodioxin-7′-yl]-3,7-dioxabicyclo[3.3.0]octane (Figure 4) [5]. HA was dissolved in dimethyl sulfoxide (DMSO) to prepare a stock solution of 10^−2^ M.

### 4.2. Insects

A *Drosophila* stock *w^1118^* was reared on standard cornmeal food at 25 °C. Third-instar larvae were selected with either left the food (“wandering” stage) or remained in the food but had reached the prominent size characteristic of wandering third-instar larvae.

### 4.3. Measurement of Synaptic Activity by Using Current Clamp

*Drosophila* larvae were dissected, and the nerves projecting from the ventral ganglion were cut. Intracellular recordings were made ventral longitudinally from muscle 6 in segments A3 and A4 of wandering third-instar larvae as previously described [35,36]. Sharp voltage-recording glass microelectrodes (20–30 MΩ) (Sutter Instrument, Novato, CA, USA), made of borosilicate glass (outer diameter, 1.2 mm), and were filled with 3 M KCl. The signal was acquired with an Axonclamp 900 A amplifier (Axon Instruments, Foster City, CA, USA), digitized with a Digidata 1440 A interface (Axon Instruments), and stored on a personal computer using pClamp 10.2 (Axon Instruments). Recordings were performed in HL3 (hemolymph-like 3) physiological solution, which produced an ionic composition and osmolarity most similar to that of *Drosophila* hemolymph [35] that contained: 70 mM NaCl, 5 mM KCl, 1.5 mM CaCl_2_, 20 mM MgCl_2_,10 mM NaHCO_3_, 5 mM trehalose, 115 mM sucrose, and 5 mM HEPES (pH was adjusted to 7.2). Only cells with a resting potential between −60 and −80 mV were included in the analysis.

Spontaneously-occurring mEJPs and evoked EJPs were recorded using sharp voltage recording. mEJPs and EJPs provided a profile of spontaneous and nerve-evoked release at the whole-cell level. Evoked EJPs were elicited by stimulating the segmental nerve at 0.1 Hz [37] with a glass-suction electrode that had been heat-polished to 10 μm inside diameter. Stimulating pulses were generated by JL-C4 V2a stimulator (Jialong Instruments, Shanghai, China). The amplitude of the pulse was set to about 5 V, which resulted in the stable recruitment of both innervating motoneurons. This result corresponded to 1.5 times the amplitude needed to recruit both motoneurons innervating muscle 6 [38].

The frequency of mEJPs was determined by counting the number of events for a period of 1 min. For each individual experiment, the frequency of mEJPs was measured three times over per minute intervals. The average of three measures was taken to represent the mEJP frequency for that particular experiment. For each mEJP and EJP average, 60 s of mEJP recording and 10 sequential EJPs were used for subsequent analysis.

The HA stock solution (10^−2^ M) was diluted in HL3 saline to prepare the test solution (10^−5^ M) at the beginning of each experiment. When the recording electrode was impaled into muscle cell and resting membrane potential was stable for 5 min, 1 mL of bath saline was drawn off from bath chamber whose bath saline volume was 4 mL, and the same volume of HL3 saline containing HA of interest was added to the chamber for recording. The final bath concentration of HA was 2.5 × 10^−6^ M at DMSO < 0.1%. Control experiments were conducted with DMSO only. Recordings were conducted as described by Peled *et al.* [28] with minor modifications. After adding HA to bath saline, a 3 min waiting period was useful for eliminating artifacts caused by Vm drift in response to fast HA application. Therefore, the recording result at 3 min after testing application was deemed the initial or baseline value for further analyses.

### 4.4. Expression of Insect Sodium Channels in Xenopus Laevis Oocytes and Electrophysiological Recording

The procedures for the functional expression and characterization of sodium channels from *Blattella germanica* (BgNa_v_), *Drosophila melanogaster* (DmNa_v_), and *Aedes aegypti* (AaNa_v_) in *Xenopus* oocytes were identical to those previously reported [39,40,41]. The procedures for oocyte preparation and cRNA injection were identical to those described previously [42]. For the robust expression of cockroach BgNa_v_ 1-1a sodium channel, *Drosophila* DmNa_v_22 channel, and *Aedes aegypti* AaNa_v_1-1 channel, their cRNAs were co-injected into oocytes with cRNA encoding the *Drosophila* *melanogaster* tipE auxiliary subunit (1:1 ratio), which enhanced the expression of insect sodium channels in oocytes.

Methods for electrophysiological recording and data analyses were identical to those described previously [42]. All experiments were performed at room temperature. Sodium currents were measured with an OC725C oocyte clamp (Warner Instruments, Hamden, CT, USA) and a Digidata 1440A interface (Axon Instruments). pCLAMP 10.2 software (Axon Instruments) was used for data acquisition and analysis.

The HA stock solution (10^−2^ M) was diluted in ND96 (consisting of 96 mM NaCl, 2.0 mM KCl, 1.0 mM MgCl2, 1.8 mM CaCl2, and 10 mM HEPES, pH adjusted to 7.5 with NaOH) recording solution as the test solution (10^−5^ M) just before experiments. DMSO concentration in the final solution was <0.5%, which had no effect on the function of sodium channels in these experiments. The method for application of chemicals in the recording system was identical to that described previously [43]. The effects of HA were measured 10 min after toxin application.

The peak current was measured by a 20 ms test pulse to −10 mV from a holding potential of −120 mV before and after HA application. The voltage dependence of sodium channel conductance (G) was calculated by measuring the peak current at test potentials ranging from −80 to +65 mV in 5-mV increments and divided by (V–Vrev), where V is the test potential and Vrev is the reversal potential for sodium ion. Peak conductance values were normalized to the maximal peak conductance (Gmax) and fitted with a two-state Boltzmann equation of the form G/Gmax = [1 + exp(*V* − *V*_1/2_)/*k*] − 1, in which *V* is the potential of the voltage pulse, *V*_1/2_ is the half-maximal voltage for activation, and k is the slope factor.

The voltage dependence of fast inactivation was determined using 200 ms inactivating prepulses from a holding potential of −120 to 40 mV in 5 mV increments, followed by test pulses to −10 mV for 20 ms. The peak current amplitude during test depolarization was normalized to the maximum current amplitude and plotted as a function of the prepulse potential. Data were fitted with a two-state Boltzmann equation of the form *I*/*I*_max_ = [1 + (exp(*V* − *V*_1/2_)/*k*)]^−1^, in which *I*_max_ is the maximal current evoked, *V* is the potential of the voltage pulse, *V*_1/2_ is the half-maximal voltage for inactivation, and *k* is the slope factor.

### 4.5. Data Collection and Analysis

Data were statistically analyzed using SPSS software (version 12.0, SPSS Inc., Chicago, IL, USA, 2003) and Microcal Origin 8.6 (Origin Lab Corp, Northampton, MA, USA, 2013). Data are presented as the mean ± S.D. Statistical significance was determined by Student’s *t*-test and Scheffe’s *post hoc* analysis, and significant values were set at *p* < 0.05 or as indicated in the table and figure legends.

## 5. Conclusions

HA could alter the response of pre- to post-synaptic transmission by delaying the decay rate of EJPs and by increasing the frequency of mEJPs at the *Drosophila* NMJ. A significant hyperpolarizing shift in the voltage-dependence of inactivation of insect sodium channels was produced. HA exerted toxic effects by disrupting the function of both conduction of action potentials and synaptic transmission. Our work provided the basis for developing a novel natural botanical pesticide based on HA as a major insecticidal active ingredient.

## Figures and Tables

**Table 1:** Voltage dependence of activation and inactivation of four sodium channels before and after the application of HA.

Na^+^ Channel Type	Activation				Inactivation			
	Toxin-Free		HA		Toxin-Free		HA	
	V_0.5_	*k*	V_0.5_	*k*	V_0.5_	*k*	V_0.5_	*k*
BgNa_v_1-1a	−31.5 ± 0.1	4.9 ± 0.1	−29.5 ± 0.2	6.1 ± 0.1	−53.5 ± 1.5	4.7 ± 0.1	−64.3 ± 1.0 *	5.4 ± 0.2
DmNa_v_22	−30.8 ± 1.9	5.3 ± 0.2	−30.5 ± 0.1	4.6 ± 0.7	−46.2 ± 0.1	5.5 ± 0.2	−54.1 ± 0.5 *	6.7 ± 0.3
AaNa_v_1-1	−30.1 ± 0.8	5.2 ± 0.5	−30.1 ± 0.7	5.7 ± 0.5	−52.8 ± 1.8	4.9 ± 0.2	−61.3 ± 0.9 *	5.6 ± 0.1

Results are the mean ± SD for 5−8 oocytes. Asterisks indicate significant differences from toxin free as determined by one-way ANOVA (*p* < 0.05) with Scheffe’s *post hoc* analysis.
